# Egg Protein Transferrin-Derived Peptides Irw (Lle-Arg-Trp) and Iqw (Lle-Gln-Trp) Prevent Obesity Mouse Model Induced by a High-Fat Diet via Reducing Lipid Deposition and Reprogramming Gut Microbiota

**DOI:** 10.3390/ijms231911227

**Published:** 2022-09-23

**Authors:** Zhuangzhuang Liu, Sujuan Ding, Hongmei Jiang, Jun Fang

**Affiliations:** 1 College of Bioscience and Biotechnology, Hunan Agricultural University, Changsha 410128, China; 2 Hunan Provincial Key Laboratory of Animal Nutritional Physiology and Metabolic Process, CAS Key Laboratory of Agro-Ecological Processes in Subtropical Region, Institute of Subtropical Agriculture, Chinese Academy of Sciences, National Engineering Laboratory for Pollution Control and Waste Utilization in Livestock and Poultry Production, Changsha 410125, China

**Keywords:** peptides, obesity, lipid determination, gut microbiota, short chain fatty acids

## Abstract

Egg-derived peptides play important roles in insulin secretion and sensitivity, oxidative stress, and inflammation, suggesting their possible involvement in obesity management. Hence, the aim of this study is to explore the alleviating effects of IRW (lle-Arg-Trp) and IQW (lle-Gln-Trp) on obesity via the mouse model induced by a high-fat diet. The entire experimental period lasted eight weeks. The results demonstrated that IQW prevented weight gain (6.52%), decreased the glucose, low-density lipoprotein (LDL), malonaldehyde, triglycerides, total cholesterol (TC), and leptin levels, and increased the concentration of adiponectin (*p* < 0.05, *n* = 8). Although IRW failed to prevent weight gain, it reduced the concentration of glucose, high-density lipoprotein (HDL), LDL, and leptin, and increased the concentration of adiponectin (*p* < 0.05, *n* = 8). Moreover, IRW and IQW increased glucose tolerance and insulin resistance based on the results of the intraperitoneal glucose test and insulin tolerance test (*p* < 0.05, *n* = 8). The quantitative polymerase chain reaction results revealed that IRW and IQW downregulated the mRNA expression of DGAT1 (Diacylglycerol O-Acyltransferase 1), DGAT2 (Diacylglycerol O-Acyltransferase 2), TNF-α, IL-6, and IL-1β of liver tissue (*p* < 0.05, *n* = 8). The results of the 16S ribosomal RNA amplicon sequencing showed that IQW and IRW tended to reduce the relative abundance of Firmicutes and Parabacteroides, and that IRW enhanced the abundance of Bacteroides (*p* < 0.05, *n* = 8). Collectively, IRW and IQW supplementation could alleviate the progression of obesity due to the fact that the supplementation reduced lipid deposition, maintained energy balance, and reprogrammed gut microbiota.

## 1. Introduction

Obesity, as a major health hazard, has attracted widespread attention due to its association with an elevated risk of death and a substantially increased risk of costly chronic diseases. A study calculated the weight and height data of 128.9 million children, adolescents, and adults from 1975 to 2018 and provided the body mass index trends for all countries in the world [1]. It warns of the rise in the prevalence of obesity in each country. Obesity is accompanied by health-threatening complications such as diabetes, hypertension, cardiovascular diseases, and stroke, thereby leading to a diminished quality of life and a shorter life expectancy [2]. The underlying cause of obesity is the energy imbalance between long-term excessive calorie intake and low calorie expenditure [3]. Enlarged adipose tissue is one of the characteristics of obesity, as it stores excess energy intake during the development of obesity, while adipocyte hypertrophy may damage adipose tissue function by inducing metabolic changes, mechanical stress, and local inflammation [4,5,6]. Although the liver is the main organ of lipid distribution, when it reaches the upper limit of lipid storage capacity, it leads to fatty liver [7]. Lipid deposition in the liver is a complex process, and the lipid secreted by the liver is essential for preventing it. Studies in rat models have shown that the main mechanism of dietary-induced hepatic steatosis is the inhibition of lipoprotein secretion [8].

Gut microbiota is a complex microbial ecosystem in the gut that plays a vital role in metabolism, immune response, and other key physiological pathways of the host [9,10,11]. The influence of gut microbiota on the host is multifaceted, including the provision of nutrients, the regulation of metabolism, and the regulation of immunity [12]. Environmental disruption of gut microbiota composition and function may cause mild inflammation, leading to obesity-related diseases [13,14,15]. Another contribution of gut microbes is the decomposition of non-digestible nutrients, including pectin, fiber, and resistant starches, which are produced by the fermentation of short-chain fatty acids (SCFAs), such as acetate, propionate, and butyrate, in the distal intestine [16]. SCFAs are an important energy source for the intestinal epithelium and liver, and they affect many important metabolic processes, including intestinal barrier function [17,18], intestinal immunity [19,20], hepatic gluconeogenesis, and adipogenesis [21,22].

Bioactive peptides play a vital role in metabolic regulation. Substantial evidence suggests that the small peptides released from the hydrolysis of animal, plant, and microbial proteins have many beneficial health properties, such as anti-hypertension [23], anti-oxidation [24], anti-obesity [25], hypocholesterolemic, and immune regulation [26,27]. IRW and IQW are identified as novel ACE inhibitory peptides that can alleviate the inflammatory response and oxidative stress of endothelial cells induced by tumor necrosis factor (TNF) [28]. These two egg protein transferrin-derived peptides were characterized from an integrated in silico digestion and comprehensive quantitative structure–activity relationship (QSAR) prediction and bioinformatics methods [29]. A study on a spontaneous hypertensive rodent model showed that IRW reduced blood pressure through the angiotensin-converting enzyme (ACE)2/Ang (1–7)/MasR axis, suggesting that IRW was the activator of ACE2 in vivo and that the activation of ACE2 was beneficial in enhancing endothelium-dependent vasodilation and reducing vascular inflammation [23]. Majumder et al. also demonstrated that IQW could not only reduce blood pressure by suppressing the generation of plasma Angiontein II, but also exert protective effects against inflammation in spontaneously hypertensive rats [30]. The purpose of the current study is to investigate whether IRW and IQW have preventive effects on the development of obesity by establishing a rodent obesity model on a high-fat (HF) diet. We explored the effects of IRW and IQW on lipid deposition, energy homeostasis, gut microbiota, and the metabolites of SCFAs, as well as the potential effect of gut microbiota on the development of obesity. The results showed that IRW and IQW could prevent obesity induced by an HF diet by reducing lipid deposition and regulating intestinal microbial composition.

## 2. Results

### 2.1. Body Weight and Organ Indexes of the Mice

**Figure 1:**
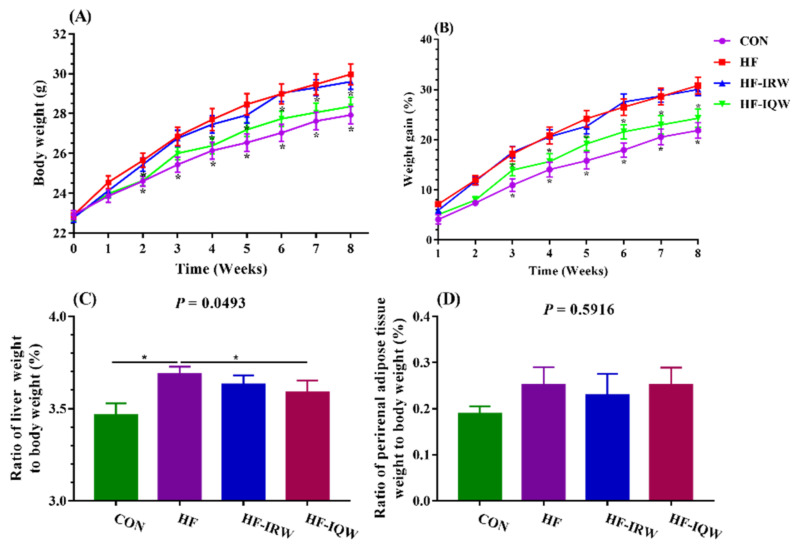
Egg protein transferrin-derived peptides IRW (lle-Arg-Trp) and IQW (lle-Gln-Trp) slowed weight gain, and IQW decreased the liver index of mice fed an HF (high-fat) diet. (**A**) Body weight of mice from week 0 to week 8; (**B**) body gain of mice from week 1 to week 8; (**C**) liver index of the mice; (**D**) perirenal adipose index of the mice. CON: the mice were fed the control diet; HF: the mice were fed an HF diet; HF-IRW: the mice were fed an HF diet and drank water supplemented with 0.03 g/L IRW starting from the fifth week; HF-IQW: the mice were fed an HF diet and drank water supplemented with 0.03 g/L IQW starting from the fifth week. * indicates *p* < 0.05 between the HF group and other groups.

The body weight and organ indexes of the mice are shown in Figure 1A–D. The results demonstrated that the body weight of the HF group remarkably increased compared to the CON group from the second week and the HF-IQW group from the fourth week (*p* < 0.05). No significant difference was observed in the HF-IRW group (*p* > 0.05). The HF diet caused a greater increase in body weight of the HF and HF-IRW groups from the second week in comparison with CON and HF-IQW groups (*p* < 0.05). IQW reduced the weight of the liver compared to the HF group (*p* < 0.05). IRW tended to decrease the weight of the liver, but no significant difference was found (*p* > 0.05). Moreover, the weight of perirenal adipose tissue showed no significant difference (*p* > 0.05). Collectively, IRW and IQW supplementation retarded weight gain.

### 2.2. Biochemical Assays, Glucose Tolerance and Insulin Aensitivity of the Mice

**Figure 2:**
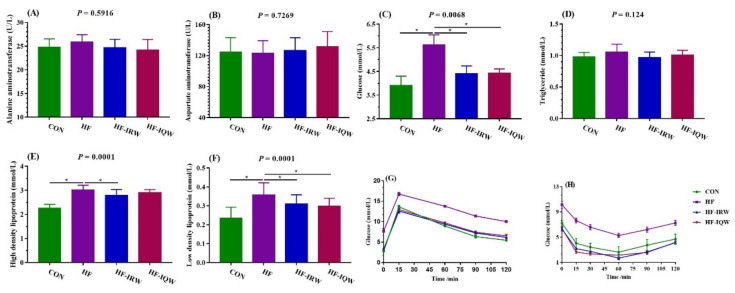
Effects of egg protein transferrin-derived peptides IRW (lle-Arg-Trp) and IQW (lle-Gln-Trp) on the biochemical serum biochemistry, GLU tolerance, and insulin sensitivity of mice fed an HF diet. (**A–F**) The biochemical analyses included alanine aminotransferase (ALT), aspartate aminotransferase (AST), glucose (GLU), triglyceride (TG), high-density lipoprotein (HDL), and low-density lipoprotein (LDL). GLU tolerance was indicated by the changing tendency of GLU after intraperitoneal glucose (**G**), and insulin sensitivity was indicated by the changing tendency of GLU after intraperitoneal insulin (**H**). * indicates *p* < 0.05 between the HF group and other groups.

The lipid and GLU levels in the serum were analyzed (Figure 2C–F). The results revealed that the HF diet increased the GLU, HDL, and LDL levels (*p* < 0.05), but it did not significantly affect the serum TG (*p* > 0.05). The GLU, HDL, and LDL levels decreased with IRW, and IQW decreased the concentrations of GLU and LDL. The serum ALT and AST levels were also measured in this study, but no significant difference was found after administration of IRW and IQW (*p* > 0.05). The IGTT and ITT results showed that the HF diet increased the GLU level (*p* < 0.05). The administration of IRW and IQW reduced the GLU level after intraperitoneal GLU and insulin. In summary, IRW and IQW improved lipid accumulation and metabolic disorders induced by an HF diet.

### 2.3. Pathological Observation and Lipid Deposition of Hepatic Tissue

**Figure 3:**
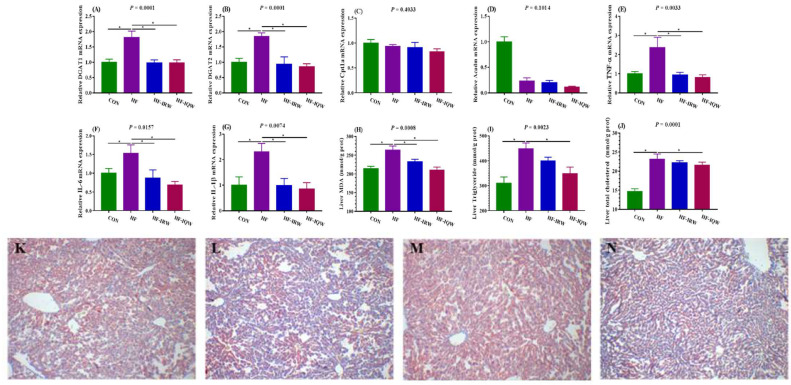
Effects of egg protein transferrin-derived peptides IRW (lle-Arg-Trp) and IQW (lle-Gln-Trp) on lipid deposition and inflammation of the liver of mice fed an HF diet. (**A**–**G**) mRNA expression of DGAT1 (Diacylglycerol O-Acyltransferase (1), DGAT2 (Diacylglycerol O-Acyltransferase (2), Cpt1a (carnitine palmitoyltransferase 1A), Acadm (acyl-CoA dehydrogenase medium chain), TNF-α, IL-6, and IL-1β. (**H**–**J**) MDA, triglyceride, and total cholesterol levels. (**K**–**N**) Oil red O staining (microscope’s magnification= 100 times) of the CON, HF, HF-IRW, and HF-IQW groups. MDA: malondialdehyde. * indicates *p* < 0.05 between the HF group and other groups.

In order to investigate the molecular mechanism of IRW and IQW in triglyceride synthesis and β-oxidation, we determined the expression of several genes in the liver tissue, such as DGAT1, DGAT2, Acadm, and Cpt1a (Figure 3A–D). The results showed that IRW and IQW decreased the mRNA expression of DGAT1 and DGAT2 (*p* < 0.05) but had no effect on the expression of Acadm and Cpt1a (*p* > 0.05). In order to analyze the hepatic inflammation response induced by an HF diet, inflammation-related genes (TNF-α, interleukin (IL)-6, and IL-1β) were measured (Figure 3E–G). The results revealed that IRW and IQW reduced the mRNA expression of TNF-α, IL-6, and IL-1β induced by an HF diet (*p* < 0.05). Moreover, we determined the oxidation products and hepatic lipid level (Figure 3H–J). The results showed that IRW and IQW reduced the hepatic MDA level (*p* < 0.05) and that IQW supplementation decreased the concentration of TG and TC (*p* < 0.05). Likewise, the results of hepatic lipid deposition measured by oil red O staining showed that IRW and IQW alleviated the lipid deposition of hepatic tissue (Figure 3K–N). These results indicate that IRW and IQW prevent the liver from undergoing an inflammation response or oxidative stress, and alleviate hepatic lipid deposition.

### 2.4. Pathological Observation and Lipid Deposition of Adipose Tissue

**Figure 4:**
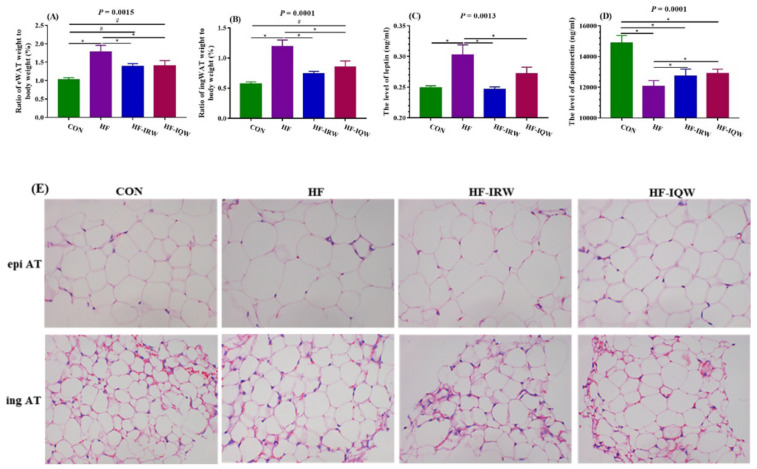
Effects of egg protein transferrin-derived peptides IRW (lle-Arg-Trp) and IQW (lle-Gln-Trp) on the lipid deposition of adipose tissue of mice fed an HF diet. (**A**,**B**) Index of ingWAT and eWAT, respectively. (**C**,**D**) Leptin and adiponectin levels. (**E**) ingWAT: inguinal white adipose tissue; eWAT: epididymal white adipose tissue (microscope’s magnification= 200 times). * indicates *p* < 0.05 between the HF group and other groups. ^#^ indicates *p* < 0.05 between the CON group and other groups.

In order to explore the energy status of adipose tissue induced by an HF diet, the weight of adipose tissue was analyzed, and the concentrations of leptin and adiponectin were measured (Figure 4A–D). The results revealed that, compared to the CON group, the HF diet increased the weight of eWAT and ingWAT as well as the leptin level, and decreased the adiponectin level (*p* < 0.05). IRW and IQW supplementation mitigated the weight of eWAT and ingWAT, decreased the concentration of leptin, and increased adiponectin induced by the HF diet (*p* < 0.05). Moreover, the HE staining results of adipose tissue demonstrated that the HF diet increased eWAT and ingWAT hypertrophy relative to the tissue in the CON group, and that IRW and IQW supplementation could retard this trend (Figure 4E). Thus, IRW and IQW could protect adipose tissue from energy disorders and tissue hypertrophy induced by an HF diet.

### 2.5. Egg Protein Transferrin-Derived Peptides IRW and IQW Can Reprogram Gut Microbiota

**Figure 5:**
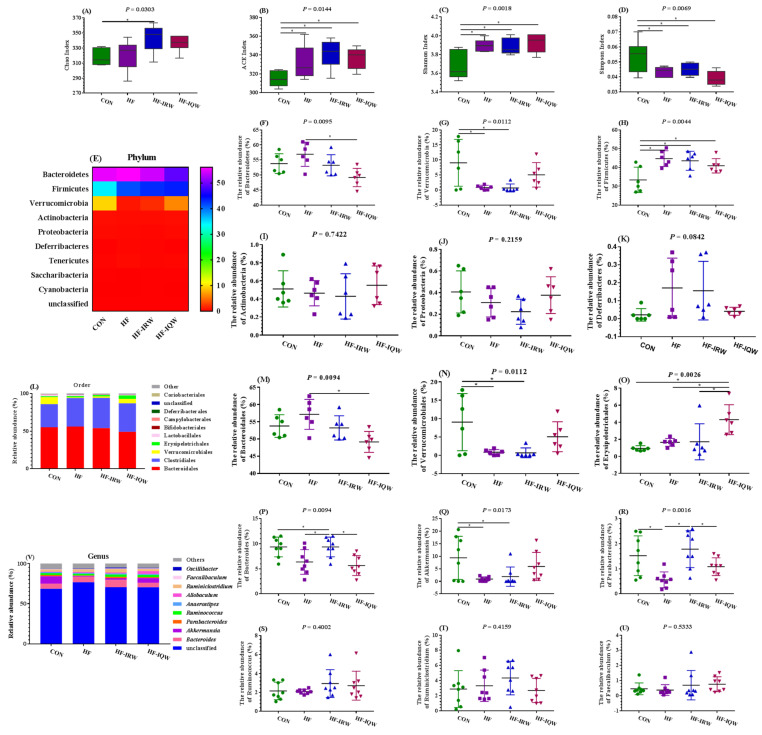
Effects of egg protein transferrin-derived peptides IRW (lle-Arg-Trp) and IQW (lle-Gln-Trp) on the gut microbiota of mice fed an HF diet. (**A**–**D**) The diverse indexes include chao index, ACE, Shannon index, and Simpson index. (**E**) Heat map of the top 10 microbiota at the phylum level. (**F**–**K**) Relative abundance of *Bacteroidetes, Verrucomicrobia, Firmicutes, Actinobacteria, Proteobacteria, and Deferribacteres*, respectively. (**L**) Microbiota composition at the order level. (**M**–**U**) Relative abundance of *Bacteroidales*, *Verrucomicrobiales, Erysipelotrichales*, *Bacteroides, Akkermansia*, *Parabacteroides*, *Ruminococcus*, *Ruminiclostridium*, and *Faecalibaculum*, respectively. * indicates *p* < 0.05. (**V**) Microbiota composition at the genus level.

Gut microbiota is closely related to obesity. Thus, the colonic microbiota was examined using 16S ribosomal RNA amplicon sequencing. The results showed that IRW and IQW increased the ACE and Shannon indexes compared to the CON group, but decreased the Simpson index (Figure 5A–D, *p* < 0.05). We further analyzed the composition of microbiota at the phylum level (Figure 5E–K) and found that IQW supplementation inhibited the growth of Bacteroidetes compared to the HF group and reduced the growth of *Verrucomicrobia* compared to the CON group. Moreover, compared to the CON group, an increased relative abundance of Firmicutes was observed in the HF diet. IRW and IQW tended to decrease the relative abundance of Firmicutes (*p* > 0.05). Moreover, IRW and IQW administration had no effect on the relative abundance of Actinobacteria, Proteobacteria, and Deferribacteres (*p* > 0.05). At the genus level, the top 10 microbiota are illustrated in Figure 5L. The results showed that *Bacteroides*, *Akkermansia*, *Parabacteroides*, and *Ruminococcus* are the main bacteria at the genus level. The HF diet decreased the relative abundance of *Bacteroidales*, *Akkermansia*, and *Parabacteroides* compared to the CON group (*p* < 0.05). IRW supplementation decreased the abundance of *Bacteroidales* and increased that of *Parabacteroides* compared to the HF group (*p* < 0.05). IQW supplementation tended to increase *Parabacteroides*, but no significant difference (*p* > 0.05) was observed. Thus, IRW and IQW supplementation could reprogram gut microbiota to maintain gut health.

**Figure 6:**
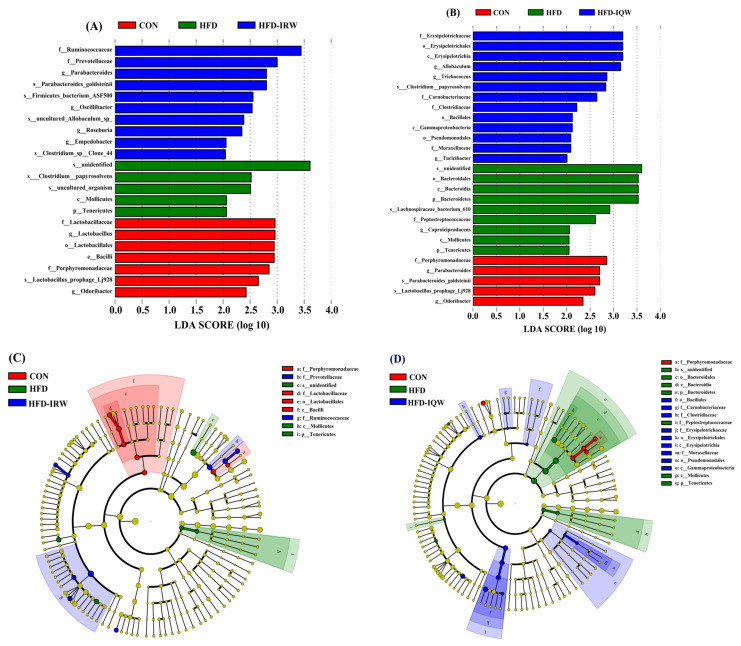
LEfSe analysis of microbiota in colon content. Linear discriminant analysis among (**A**) and (**C**) CON, HFD, and HFD-IRW, (**B**) and (**D**) among CON, HFD, and HFD-IQW. The red, green, and blue nodes in the phylogenetic tree signify the microbial species which perform a vital role in the groups. The yellow nodes signify the species with no significant difference.

The LEfSe analysis of colonic microorganisms among CON, HFD, and HFD-IRW groups is shown in Figure 6A. The result revealed that four bacterial biomarkers were detected in the CON group, including Porphyromonadaceae, Lactobacillaceae, Lactobacillales and Bacilli; two bacterial biomarkers were detected in the HFD group, including Mollicutes and Tenericutes; and two bacterial biomarkers were detected in the HFD-IRW group, including Prevotellaceae and Ruminococcaceae. The LEfSe analysis of colonic microorganisms among CON, HFD, and HFD-IQW groups is shown in Figure 6B. The result revealed that one bacterial biomarker was detected in the CON group (Porphyromonadaceae); six bacterial biomarkers were detected in the HFD group, including Bacteroidales, Bacteroidia, Bacteroidetes, Peptostreptococcaceae, Mollicutes and Tenericutes; and nine bacterial biomarkers were detected in the HFD-IQW group, including Bacillales, Carnobacteriaceae, Clostridiaceae, Erysipelotrichaceae, Erysipelotrichales, Erysipelotrichia, Moraxellaceae, Pseudomonadales, Gammaproteobacteria.

### 2.6. SCFA Concentration in Feces

**Figure 7:**
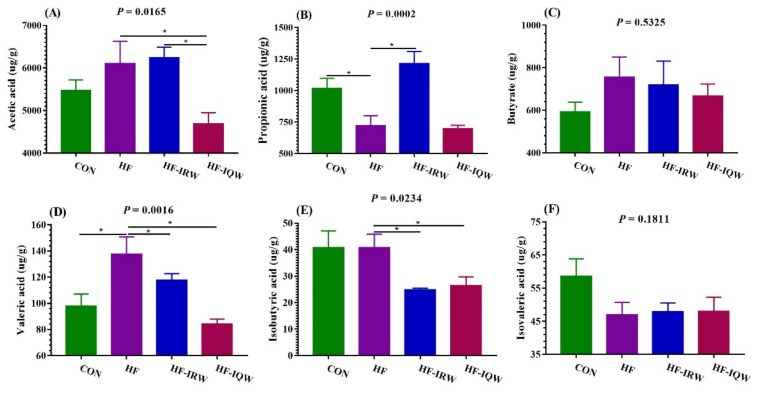
Effects of egg protein transferrin-derived peptides IRW (lle-Arg-Trp) and IQW (lle-Gln-Trp) on SCFAs (short-chain fatty acid) in the feces of mice fed an HF diet. (**A**) Concentration of acetic acid; (**B**) concentration of propionic acid; (**C**) concentration of butyrate; (**D**) concentration of Valeric acid; (**E**) concentration of Isobutyric acid; (**F**) concentration of Isovaleric acid. * indicates *p* < 0.05 between the HF (high-fat diet) group and other groups.

SCFAs have many beneficial effects on the mammalian energy metabolism. To examine the change in SCFAs in feces, we measured their concentrations (Figure 7A–F). The HF diet was found to decrease the level of propionic acid and increase the level of valeric acid compared to the CON group (*p* < 0.05). IRW and IQW supplementation decreased the concentration of valeric acid and isobutyric acid relative to the HF group (*p* < 0.05). Moreover, IQW supplementation decreased the acetic acid level compared to the HF group, and IRW supplementation increased the propionic acid level compared to the HF group (*p* < 0.05).

### 2.7. Correlation Analysis between Differential Gut Microbe with SCFA or Lipid Deposition-Related Factors in Serum

**Figure 8:**
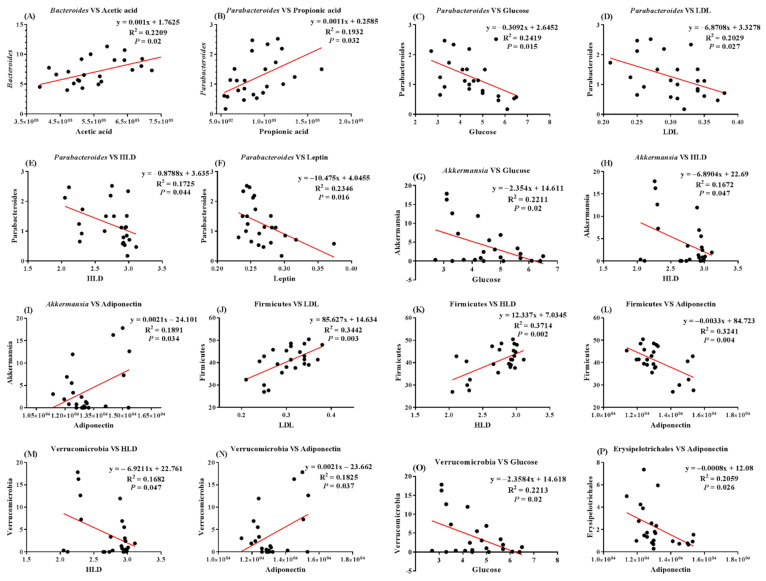
Correlation analysis between the gut microbe with SCFAs (short-chain fatty acids) and the lipid deposition-related factor. (**A**) *Bacteroides* vs. acetic acid; (**B**) *Parabacteroides* vs. propionic acid; (**C**) *Parabacteroides* vs. GLU; (**D**) *Parabacteroides* vs. LDL; (**E**) *Parabacteroides* vs. HLD; (**F**) *Parabacteroides* vs. leptin; (**G**) *Akkermansia* vs. GLU; (**H**) *Akkermansia* vs. HLD; (**I**) *Akkermansia* vs. adiponectin; (**J**) *Firmicutes* vs. LDL; (**K**) *Firmicutes* vs. HLD; (**L**) *Firmicutes* vs. adiponectin; (**M**) *Verrucomicrobia* vs. HLD; (**N**) *Verrucomicrobia* vs. adiponectin; (**O**) *Verrucomicrobia* vs. GLU; (**P**) *Erysipelotrichales* vs. adiponectin.

Gut microbiota affects host nutrition and energy regulation, as well as the development of obesity. In order to explore the relationship between gut microbiota and other chemical substances in the development of obesity, we investigated the correlation between gut microbiota with SCFA and lipid deposition-related factors (Figure 8A–P). The results showed a positive correlation between acetic acid and *Bacteroides*; a positive correlation between propionic acid and *Parabacteroides*; a negative correlation between GLU and *Parabacteroides*/*Akkermansia*/*Verrucomicrobia*; a negative correlation between LDL and *Parabacteroides,* but a positive correlation with *Firmicutes*; a negative correlation between HLD and *Parabacteroides/Akkermansia/Verrucomicrobia,* but a positive correlation with Firmicutes; a negative correlation between leptin and *Parabacteroides*; and a positive correlation between adiponectin and *Akkermansia*/*Verrucomicrobia,* but a negative correlation with *Firmicutes* and *Erysipelotrichales*.

## 3. Discussion

As multifunctional compounds derived from proteins, bioactive peptides have a positive effect on body functions and may ultimately affect health [31]. Most natural processes in the body are signaled or regulated by the interaction of specific amino acid sequences, which can be peptide or protein segments [32]. The amino acid sequence of bioactive peptides may affect major body systems, such as hypertension, diabetes, cardiovascular, antimicrobial, and immune, and therefore have broad therapeutic applications in the future [23,33]. The milk-derived peptide Val-Pro-Pro was found to prevent a fatty inflammation response between fat cells and macrophages and to act as an ACE inhibitor to improve obesity-related insulin resistance, thereby improving the development of obesity [34]. In addition, studies have shown that IRW reduces blood pressure in spontaneously hypertensive rats, acts as an ACE2 activator to enhance endothelium-dependent vasodilation, and reduces vascular inflammation [23]. Our study demonstrated that IRW and IQW could reduce lipid deposition, increase GLU tolerance and insulin resistance, and reprogram gut microbiota.

WAT is a loose connective tissue with a highly organized vascular system. In addition to the functions of structural buffering, passive insulation, and GLU and lipid homeostasis, WAT also has other functions, such as regulating the metabolism and immune function of endocrine signaling organs [35,36]. Lipid homeostasis and immune function in the body may be out of order after excess adiposity caused by adipocyte hypertrophy and hyperplasia. Excess lipids and impaired lipid storage are partly responsible for obesity; thus, fatty acids affect insulin signaling pathways [37]. Adipose tissue is the main source of energy for the human body, along with adipocytokine adiponectin and leptin [38]. Leptin is an anti-obesity hormone secreted by adipocytes discovered through positional cloning, and it has pleiotropic effects on energy homeostasis as well as endocrine and metabolic physiology and pathology [39]. Leptin levels in human plasma are usually associated with fat mass and changes in energy [40]. Adiponectin, the protein most abundant in WAT, has been implicated in the regulation of insulin resistance, type 2 diabetes, atherosclerosis, and other diseases induced by obesity [41]. Our results showed that IRW and IQW supplementation inhibited the hypertrophy of eWAT and ingWAT and reduced the leptin and adiponectin levels induced by the HF diet (Figure 4A–D).

Hepatic lipid accumulation may lead to fatty liver and contribute to systemic metabolic dysfunction. The severity of fatty liver disease is directly relevant to the characteristics of metabolic syndrome, including hyperglycemia, insulin resistance, hypertriglyceridemia, and hyperinsulinemia [42,43]. An HF diet has been found to induce hepatomegaly and lipid deposition and to increase triglyceride levels in rodent models [44]. The liver plays an important role in maintaining GLU homeostasis. In insulin resistance, an increase in gluconeogenesis leads to an increase in hepatic glucose production [45,46]. Moreover, the disorder of glucose metabolism regulation is one of the characteristics of obesity, and is often accompanied by elevated levels of chronic inflammatory markers in the liver and obese tissues, such as TNF-α, IL-6, and IL-1 β [47]. An uncontrolled proinflammatory response may contribute to a chronic inflammatory state, promote a favorable tumor microenvironment, or promote immune transition activation and cancer growth [48]. Our results suggest that IRW and IQW reduced the levels of glucose, HDL, and LDL, improved GLU tolerance and insulin resistance induced by the HF diet, and decreased the mRNA expression of TNF-α, IL-6, and IL-1β (Figure 3E–G). Moreover, we examined the expression of Cpt1a and Acadm, which participated in β-oxidation, as well as the expression of DGAT1 and DGAT2, which participate in triglyceride synthesis. IRW and IQW promoted the expression of DGAT1 and DGAT2 in liver tissue and reduced the concentration of serum MDA, but they had no significant effect on Cpt1a and Acadm (Figure 3A–G).

Unhealthy eating habits have an adverse effect on gut microbiota homeostasis, which can lead to low levels of inflammation, thereby inducing diseases associated with obesity [12]. The variation in gut microbiota controls metabolic endotoxemia, inflammation, and related diseases by increasing intestinal permeability. The level of LPS in the cecum was reduced in *ob/ob* mice, and the mice were fed an HF diet after administering antibiotics. This effect has also been found to be associated with reduced GLU intolerance, weight gain, and inflammation. In addition, better intestinal permeability was shown in the HF diet, but the expression of genes related to the tight junction protein in intestinal tissue was inhibited [49]. Generally, obese individuals had decreased Bacteroidetes and increased Firmicutes levels (Figure 5F–K) [50]. However, changes at the smaller microbial community level, rather than at the phylum level, have been found to be associated with the development of obesity [51,52]. *Parabacteroides distasonis* (PD) is a probiotic in the human body that treats diarrhea and constipation. Its content is negatively correlated with obesity, non-alcoholic fatty liver disease, and diabetes. One study revealed that PD improved the symptoms of obesity, insulin resistance, lipid metabolism disorder, and non-alcoholic fatty liver in HF diet-fed mice and *ob/ob* model mice, which could be related to the extensive bile acid conversion and intestinal gluconeogenesis function of PD [53]. In addition, Bacteroides, as the main microorganism in the intestine, has anti-obesity effects in animal models [54]. Our results show that IQW and IRW tended to decrease the relative abundance of *Firmicutes* and *Parabacteroides,* and that IRW increased the abundance of *Bacteroides*. The mechanism of egg protein transferrin-derived peptides regulating fat deposition based on microorganisms needs further exploration.

## 4. Methods and Materials

### 4.1. Animal and Experimental Design

An animal experiment was conducted in accordance with Hunan Agricultural University’s rules for the care and use of laboratory animals, with approval from the animal care committee of Hunan Agricultural University. A total of 32 nine-week-old C57BL/6J male mice were obtained from SLAC Laboratory Animal Central (Changsha, China).

Egg protein transferrin-derived peptides were synthesized by ChinaPeptides (Suzhou, Jiangsu, China). Purity detection of freeze-dried peptides was conducted by high performance liquid chromatography. The purity of IRW (Molecular Weight: 473.58) and IQW (Molecular Weight: 445.52) was 87.91% and 93.04%, respectively, and impurity includes moisture and water-soluble salt.

All animals were bred in a sterile environment at a room temperature of 22 ± 2 °C, relative humidity of 50 ± 5%, light cycle of 12 h/d (i.e., 6:30 a.m. to 6:30 p.m.), and with ad libitum food and water. After seven days of acclimation, all animals were randomly divided into four groups (Table 1, *n* = 8). The HF diet consisted of 54.9% corn, 5.6% casein, 18% soybean, 6.5% brewer’s yeast, 11.3% lard, 0.8% soybean oil, 0.5% salt, 1.4% fish meal, and 1% premix. In accordance with Research Diets, the lard content in the control diet was 0.7%. The entire experimental period lasted eight weeks. During the last week of the experiment, after collecting blood from the retroorbital sinus, each mouse was sacrificed through cervical dislocation. Afterward, the liver and white adipose tissue (WAT), including epididymal white adipose tissue (eWAT), inguinal white adipose tissue (ingWAT), and perirenal adipose tissue, were separated and collected. The liver and adipose tissue were fixed in a 4% formaldehyde solution. The colon contents were kept in sterile tubes. The sample was snap-frozen in liquid nitrogen and then stored at −80 °C until analysis.

The intraperitoneal glucose test (IGTT) and insulin tolerance test (ITT) were carried out nine days and seven days before the end of the experiment, respectively. After 6 h of fasting, the mice were injected intraperitoneally with 1 g/kg body weight of glucose, and blood glucose concentrations was detected through the tail vein at 0, 15, 60, 90, and 120 min. The mice were injected intraperitoneally with 0.65 U/kg body weight of insulin, and blood glucose concentrations were measured at 0, 15, 30, 60, 90, and 120 min.

### 4.2. Biochemical Assays

The biochemical indicators measured in serum included alanine aminotransferase (ALT), aspartate aminotransferase (AST), glucose (GLU), triglyceride (TG), high-density lipoprotein (HDL), and low-density lipoprotein (LDL), following the manufacturer’s instructions (Nanjing Jiancheng Bioengineering Institute, Nanjing, China). The levels of leptin and adiponectin were detected according to the manufacturer’s instructions (CUSABIO, Wuhan, China).

### 4.3. Detection of Triglyceride (TG), Total Cholesterol (TC) and Malondialdehyde (MDA) in Mice of Liver Tissue

The liver tissues were homogenized at 1:9 (m:v) and centrifuged at 6000 rpm for 10 min at 4 °C before transferring the supernatant for further detection. TG, TC, and MDA levels were measured following the instructions mentioned previously (Jiancheng Bioengineering Institute). The concentration of protein was determined according to the BCA Protein Assay Kit (Beyotime, Shanghai, China).

### 4.4. Pathological Observation of Liver and Adipose Tissue

The liver was immobilized in 4% formaldehyde for 48 h. It was cut into 8-μm thick slices, which were roasted, dewaxed, and stained with hematoxylin and eosin (HE) for 1 min. The slices were dehydrated using a gradient concentration of alcohol and sealed with neutral gum. Liver tissues of pathological status were observed using an optical microscope (Motic, Beijing, China).

### 4.5. Oil Red O Stain and Hepatic Lipid Determination

The frozen embedded liver tissues were cut into 6-μm thick slices, and 100 μL oil red O was added. The slices were placed in a wet box, incubated at room temperature for 20 min, and observed under a microscope (Motic, Beijing, China) after hematoxylin staining and buffered glycerin sectioning.

### 4.6. Quantitative Polymerase Chain Reaction (PCR) Analysis

Total RNA from the liver was extracted using TRIZOL reagent (Invitrogen, Carlsbad, CA, USA) and then treated with DNase I (Invitrogen). The reverse transcriptional program was performed at 37 °C for 15 min and at 95 °C for 5 s. The primers used in this study are shown in Table S1. β-actin served as the housekeeper gene for gene normalization. The PCR cycling program was conducted at 94 °C for 5 s and at 5 °C and 72 °C for 30 s. The target gene of the relative expression in the comparison with β-actin was calculated using the method of comparing the Ct value.

### 4.7. 16S rRNA Amplicon Sequencing for Colon Microbe

The process of 16S rRNA sequencing for the colon microbe was performed following a previous study [55]: genomic DNA quality control, design, and synthesis of the primer splice, PCR amplification and purification of PCR products, quantification and homogenization of PCR products, and MiSeq high-throughput sequencing (Illumina, San Diego, CA, USA).

### 4.8. Determination of Faecal Short-Chain Fatty Acid

The concentration of SCFAs and other related organic acids (acetic acid, butyrate, valeric acid, isovaleric acid, etc.) was analyzed using a gas chromatography (GC) system (Agilent 7890A, Agilent, Palo Alto, CA, USA). The GC detection program parameters are shown in the supplementary materials. A freeze-dried colon digesta of 100 mg was mixed with 1 mL of ultrapure water and centrifuged at 10,000 rpm for 10 min at 4 °C. The supernatant was adjusted to 1 mL. Then, 25% metaphosphoric acid was added at a 9:1 ratio: a 900 μL sample and 100 μL of 25% metaphosphoric acid (Sinopharm, Shanghai, China) were mixed and added into a 2 mL tube. After mixing, the mixture was left to sit at a standing reaction time of 3–4 h at an ambient temperature. The mixture was then centrifuged at 12,000 rpm for 15 min. The supernatant was filtered using a 45-μm microporous filter membrane (nylon series) and then added to the sample bottle for on-board detection. 

### 4.9. Data Analysis

Data analysis was conducted using a one-way analysis of variance to check the homogeneity of variance, using Levene’s test and Student’s test through SPSS 21 (Chicago, IL, USA) for Windows. GraphPad Prism 7.00 (San Diego, CA, USA) software was used to conduct a Pearson correlation between the colonic microbe with SCFA and the lipid deposition-related factors in the serum. A significant difference was determined at *p* < 0.05.

## 5. Conclusions

This study proved that IQW and IRW exhibited a mitigative effect on HF diet-induced obesity in a rodent model. The results showed that IQW and IRW ameliorated the negative effect of the HF diet on GLU tolerance and insulin resistance, reduced hepatic lipid deposition and inflammation, improved the hypertrophy of adipose tissue, and reprogrammed gut microbiota. Moreover, IQW prevented weight gain, but IRW did not. A Pearson correlation analysis found that *Parabacteroides* was negatively correlated with GLU, LDL, and leptin, but positively correlated with propionic acid. This finding is consistent with previous studies in that the abundance of *Parabacteroides* is negatively correlated with obesity. Conversely, Firmicutes was positively correlated with LDL and HDL, but negatively correlated with adiponectin. This suggests that *Firmicutes* may be related to lipid deposition and energy regulation. However, more research is needed to confirm the potential role of these bacteria in obesity regulation, and to provide more evidence for egg protein transferrin-derived peptides IRW and IQW in alleviating the adverse effects of obesity.

## Figures and Tables

**Table 1:** Group name and treatment.

Group Name	Treatment
Group 1, control group: CON	mice were fed the control diet
Group 2, high-fat diet: HF	mice were fed a high-fat diet
Group 3, HF-IRW (lle-Arg-Trp)	mice were fed a high-fat diet and drank water supplemented with 0.03 g/L IRW starting from the fifth week
Group 4, HF-IQW (lle-Gln-Trp)	mice were fed a high-fat diet and drank water supplemented with 0.03 g/L IQW starting from the fifth week

## Data Availability

The raw sequence data in this study are uploaded in the NCBI database, the accession is PRJNA853190 (http://www.ncbi.nlm.nih.gov/bioproject/?term=PRJNA853190) and https://www.ncbi.nlm.nih.gov/Traces/study/?acc=PRJNA853190&o=acc_s%3Aa.

## Supplementary Materials

The supporting information can be downloaded at: https://www.mdpi.com/article/10.3390/ijms231911227/s1.
