# Supplementary material for: Egg Protein Transferrin-Derived Peptides Irw (Lle-Arg-Trp) and Iqw (Lle-Gln-Trp) Prevent Obesity Mouse Model Induced by a High-Fat Diet via Reducing Lipid Deposition and Reprogramming Gut Microbiota
Source: Int J Mol Sci. 2022 Sep 23;23(19):11227. doi: 10.3390/ijms231911227 (PMC9569728; doi:10.3390/ijms231911227)
Supplement: Supplementary file 1 [file ijms-23-11227-s001.zip › ijms-1909544-supplementary.pdf]

Supplementary materials

Table S1. Primers used in this study.

Gene	5’-3’ Primer sequence
β-actin	F: TGTCCACCTTCCAGCAGATGT<br>R: AGCTCAGTAACAGTCCGCCTAGA
DGAT1	F: TTCCGCCTCTGGGCATT<br>R: AGAATCGGCCCACAATCCA
DGAT2	F: AGTGGCAATGCTATCATCATCGT<br>R: TCTTCTGGACCCATCGGCCCCAGGA
Cpt1a	F: CAGTCGACTCACCTTTCCTG<br>R: CATCATGGCTTGTCTCAAGTG
Acadm	F: TGCTCGCAGAAATGGCGATGA<br>R: CAATGTGCTCACGAGCTATGA
TNF-α	F: ATGAGAAGTTCCCAAATGGC<br>R: CTCCACTTGGTGGTTTGCTA
IL-6	F: CCTCTCTGCAAGAGACTTCCAT<br>R: AGTCTCCTCTCCGGACTTGT
IL-1β	F: TGCCACCTTTTGACAGTGATG<br>R: AAGGTCCACGGGAAAGACAC

The GC system detection program:

Nitrogen as carrier gas entered the detection system at a velocity of 40 mL/min. The initial temperature of the oven was 60°C and kept for 2 min, and then the temperature rose to 220°C at 20°C/min and hold for 3.5 min. The temperature of FID and inlet temperature was 260°C and 250°C respectively. The flow rates of hydrogen and air as fuel gas and oxidizer gas were 30 mL/min and 350 mL/min, respectively. The injection volume was 1 μl.
